# Comparison of the toxicokinetics of the convulsants picrotoxinin and tetramethylenedisulfotetramine (TETS) in mice

**DOI:** 10.1007/s00204-020-02728-z

**Published:** 2020-04-01

**Authors:** Brandon Pressly, Natalia Vasylieva, Bogdan Barnych, Vikrant Singh, Latika Singh, Donald A. Bruun, Sung Hee Hwang, Yi-Je Chen, James C. Fettinger, Stephanie Johnnides, Pamela J. Lein, Jun Yang, Bruce D. Hammock, Heike Wulff

**Affiliations:** 1 Department of Pharmacology, Genome and Biomedical Sciences Facility, University of California, Room 3502, 451 Health Sciences Drive, Davis, CA 95616 USA; 2 Department of Entomology and Nematology, and Comprehensive Cancer Center, University of California, Davis, USA; 3 Department of Molecular Biosciences, University of California, Davis, USA; 4 Department of Chemistry, University of California, Davis, USA; 5 City University Veterinary Medical Centre, Sham Shui Po, Hong Kong

**Keywords:** TETS, GABA_A_ receptor, Picrotoxinin, Convulsant, Threat agent

## Abstract

Acute intoxication with picrotoxin or the rodenticide tetramethylenedisulfotetramine (TETS) can cause seizures that rapidly progress to *status epilepticus* and death. Both compounds inhibit γ-aminobutyric acid type-A (GABA_A_) receptors with similar potency. However, TETS is approximately 100 × more lethal than picrotoxin. Here, we directly compared the toxicokinetics of the two compounds following intraperitoneal administration in mice. Using LC/MS analysis we found that picrotoxinin, the active component of picrotoxin, hydrolyses quickly into picrotoxic acid, has a short in vivo half-life, and is moderately brain penetrant (brain/plasma ratio 0.3). TETS, in contrast, is not metabolized by liver microsomes and persists in the body following intoxication. Using both GC/MS and a TETS-selective immunoassay we found that mice administered TETS at the LD_50_ of 0.2 mg/kg in the presence of rescue medications exhibited serum levels that remained constant around 1.6 μM for 48 h before falling slowly over the next 10 days. TETS showed a similar persistence in tissues. Whole-cell patch-clamp demonstrated that brain and serum extracts prepared from mice at 2 and 14 days after TETS administration significantly blocked heterologously expressed α_2_β_3_γ_2_ GABA_A_-receptors confirming that TETS remains pharmacodynamically active in vivo*.* This observed persistence may contribute to the long-lasting and recurrent seizures observed following human exposures. We suggest that countermeasures to neutralize TETS or accelerate its elimination should be explored for this highly dangerous threat agent.

**Electronic supplementary material:**

The online version of this article (10.1007/s00204-020-02728-z) contains supplementary material, which is available to authorized users.

## Introduction

Tetramethylenedisulfotetramine (TETS or tetramine) is a synthetic rodenticide that is currently banned worldwide and listed as a potential threat agent by the United States Department of Homeland Security because of its extreme toxicity (Laukova et al. 2019b). TETS is odorless and tasteless; the lethal dose in adult humans is 7–10 mg (Guan et al. 1993). Due to its high toxicity and its persistence in the environment (Whitlow et al. 2005), TETS never became commercially available in the United States. However, because of its exceptional efficacy against rodents, TETS continues to be widely used in China (Thundiyil et al. 2008; Yong 2018). In addition to accidental human exposures, TETS has also been employed with malicious intent. Between 1991 and 2010 a total of 14,000 intoxications with 932 deaths, mostly from adulterated food or drink, have occurred in China (Li et al. 2012, 2014). There also have been cases of reported TETS poisonings in the United States with material sourced from China (Barrueto et al. 2003; Whitlow et al. 2005). In contrast to the synthetic TETS, picrotoxin is an equimolar mixture of two tricyclic sesquiterpenes: picrotin and the active component picrotoxinin (Slater and Wilson 1951). Picrotoxin has an extremely bitter taste, hence its name, the “bitter” poison and has been used as an antidote for barbiturate overdosing between 1930 and 1960 (Sita-Lumsden 1949; Wax 1997). Today, picrotoxin or its active component picrotoxinin, are mostly used as pharmacological tools to inhibit γ-aminobutyric acid type A (GABA_A_) receptor-mediated chloride currents (Ffrench-Constant et al. 1993; Olsen 2015). While picrotoxinin shows little subtype selectivity and inhibits most recombinantly expressed GABA_A_ receptors with an IC_50_ of roughly 4 μM in whole-cell patch-clamp experiments, TETS inhibits α_2_β_3_γ_2_ (IC_50_ 480 nM) and α_6_β_3_γ_2_ (IC_50_ 400 nM) receptors 7–10-fold more potently than most other GABA_A_ receptors (Pressly et al. 2018). Since α_2_β_3_γ_2_ receptors make up 15–20% of the GABA_A_ receptors in the mammalian brain (Fritschy and Mohler 1995; Pirker et al. 2000; Rudolph and Knoflach 2011), we previously proposed that α_2_β_3_γ_2_ is probably the most important GABA_A_ receptor for the seizure-inducing activity of TETS (Pressly et al. 2018).

**Fig. 1:**
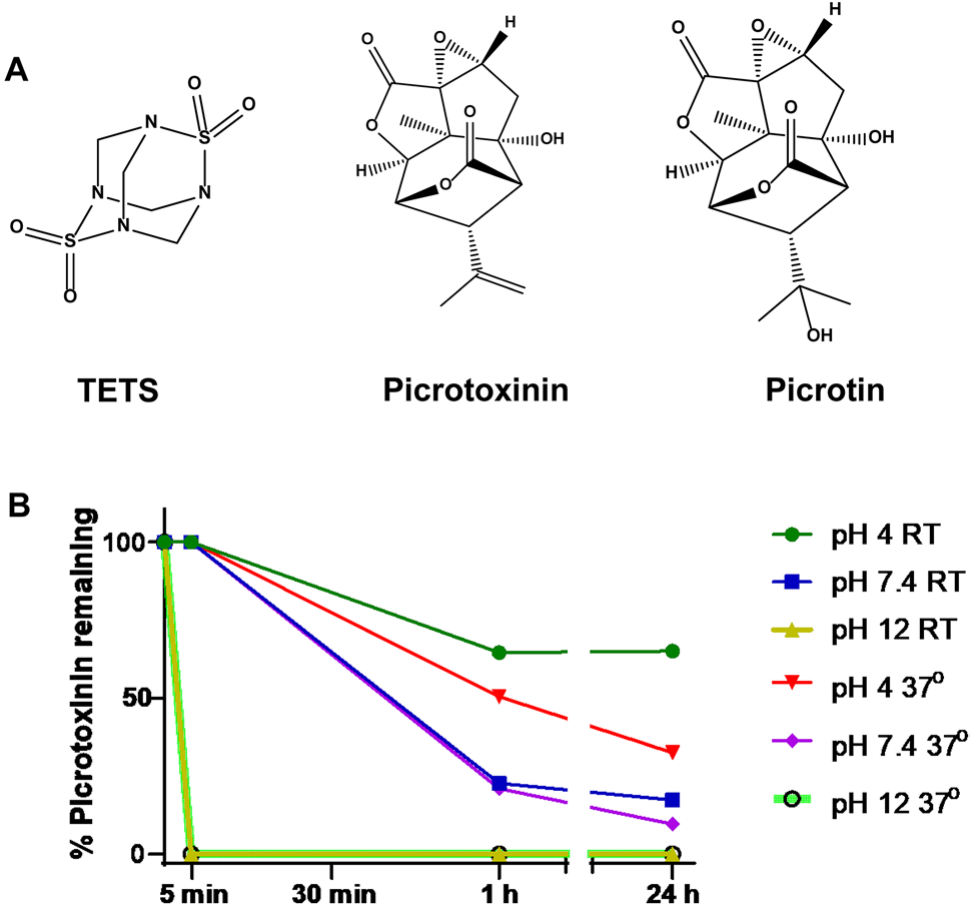
**a** Chemical structures of TETS, picrotoxinin, and picrotin. **b** Stability of picrotoxinin at pH 4, pH 7.4 and pH 12 at room temperature and at 37 °C

Acute intoxication with either picrotoxin or TETS (Fig. 1), can trigger seizures, which depending on the dose, rapidly progress to death in rodents or humans (Li et al. 2012; Whitlow et al. 2005; Zolkowska et al. 2012). However, while picrotoxin has never been reported to induce any significant long-term sequelae such as cognitive dysfunction, or spontaneous recurrent seizures (SRS) when it was used in humans as a drug and later as a pharmacological tool in rodents, these types of long-term morbidities are frequently observed in human survivors of TETS poisoning (Laukova et al. 2019b; Li et al. 2012; Whitlow et al. 2005). In this context, it is currently not clear whether TETS is more epileptogenic than other GABA_A_ antagonists, or whether it indeed persists in the body as suggested by a pharmacokinetic study in rabbits (Zhang et al. 2005). The latter observation is at variance with a radiotracer study from 1970 that suggested relatively rapid metabolism and excretion (Radwan 1970). Another fact that puzzled us is that the reported seizure-inducing and lethal doses for picrotoxin, roughly 50% of which is the active picrotoxinin, vary so widely in the literature. Picrotoxin doses administered intraperitoneally to induced seizures in animal models range from as low as 0.1 mg/kg to doses as high as 30 mg/kg in mice or rats (Cymerblit-Sabba and Schiller 2010; Klaassen et al. 2006), but average between 3–10 mg/kg (Makinson et al. 2014; Shimshoni et al. 2008). The reported LD_50_s vary from 3 to 50 mg/kg (Pericic and Bujas 1997; Pericic et al. 2000; Szabo et al. 1984). In contrast, the reported LD_50_ values of TETS are remarkably consistent in the literature. Irrespective of whether the administration route is oral, subcutaneously or intraperitoneally, the LD_50_ in mice or rats is 0.1–0.2 mg/kg with a steep dose–response relationship typically achieving 100% lethality around 0.4 mg/kg (Laukova et al. 2019a; Rice et al. 2017; Shakarjian et al. 2012; Zolkowska et al. 2012). Juvenile rats of both sexes are slightly more sensitive to TETS than adult animals (Laukova et al. 2018).

To understand these differences between picrotoxinin and TETS and inform the development of countermeasures against TETS, we here directly compared the toxicokinetics of TETS and picrotoxinin in mice at doses approximately corresponding to the LD_50_.

## Materials and methods

### Chemicals

TETS, [^13^C]-TETS and [^14^C]-TETS were synthesized as previously described (Zhao et al. 2014). Picrotoxinin was purchased from MilliporeSigma. Both TETS and picrotoxinin waste was treated with concentrated nitric acid and disposed of using the waste accumulation program at University of California, Davis. Riluzole was purchased from Oakwood Products (West Columbia, SC) and recrystallized in our laboratory to increase purity to 98%. ^1^H and ^13^C nuclear magnetic resonance were used to confirm identity and purity. Purity of all compounds used in this study was > 98%. Midazolam was purchased as pharmaceutical grade (5 mg/mL midazolam in 0.8% sodium chloride, 0.01% edetate disodium, 1% benzyl alcohol) from Hospira, Lake Forest, IL.

### Stability study of picrotoxinin

Stock solutions of picrotoxinin (100 mM) were prepared in dry DMSO and diluted to 50 μM (picrotoxinin) into phosphate-buffered saline (PBS) or water (Optima^®^, LC/MS grade, Fisher Chemical) at pH 4.0, 7.4 and 12.0 (1 mL volume in clear 4-mL glass vials) and kept either at room temperature or at 37 °C. Stability was tested at 5 min, 1 h and 24 h.

### Animals

Male NIH-NCI (National Institute of Health, National Cancer Institute) Swiss mice (Strain code: 550) were purchased from Charles River Laboratories at 12–14 weeks of age and maintained in facilities fully accredited by the Association for Assessment and Accreditation of Laboratory Animal Care. All animals were acclimated to the new vivarium for at least 7 days. Experiments were carried out in accordance with the Guide for the Care and Use of Laboratory Animals (NIH Publication No. 8023) and approved by the Institutional Animal Care and Use Committee of the University of California, Davis.

### Picrotoxinin plasma protein binding

Picrotoxinin plasma protein binding was determined at a concentration of 2.5 μM using rapid equilibrium devices (RED, Fisher Scientific) according to the manufacturer’s protocol at 37 °C. First, the time required to reach equilibrium was determined in PBS buffer (pH 7.4) by testing at 30 min, 1 h, 2 h, 3 h and 4 h. Equilibrium distribution between the chambers was reached in 1 h. Spiked plasma samples (*n* = 4) were run for 1 h to reach equilibrium. In parallel, picrotoxinin protein binding was also determined with ultracentrifugation. Using Amicon Ultra 0.5 mL 30 K filters (MilliporeSigma) each filter was spun at 16,873 × g to elute for just 10 min to minimize picrotoxinin degradation during the experiment.

### Picrotoxinin pharmacokinetics

For intraperitoneal application, 2 mg of picrotoxinin was dissolved in 10 mL of 1:4 DMSO/PBS. Previously reported vehicles for picrotoxin (Zolkowska et al. 2012) were found to leave significant amounts of compound undissolved. A total of 42 adult male NIH-NIC Swiss mice (16 weeks, 21–34 g) were then dosed with 2 mg/kg picrotoxinin in three separate cohorts on different days. Following dosing, mice were closely monitored and seizure behavior scored based on the Racine score (Racine 1972). Mice started to exhibit reduced motility (score of 1) 10 min after the intraperitoneal injection. At all other time points, mice showed myoclonic jerks (score of 3) as a minimum or loss of righting reflex (score of 4) and regularly had tonic seizures (score of 5). If death occurred (overall lethality was 25%), it typically occurred between 20–30 min post exposure while mice were seizing. Mice surviving the initial seizures typically survived until sacrifice. Mice were anesthetized with 5% isoflurane and euthanized by exsanguination. At 10, 20, 30, 60 min, 2 h, or 3 h post picrotoxinin administration brain, liver and blood from the vena cava were obtained (*n* = 3–8 per time point and tissue). Blood was treated with powdered EDTA and spun for 10 min at 8,608 × g and the resulting plasma was immediately processed for LC/MS analysis.

### Picrotoxinin LC/MS analysis

A 5-mM stock solution of picrotoxinin was prepared freshly every day by dissolving 5.1 mg picrotoxinin in 3.49 mL acetonitrile (Optima^®^, LC/MS grade, Fisher Chemical). Working standard solutions were obtained by diluting the stock with acetonitrile. [Please note that picrotoxinin readily decomposes in nucleophilic solvents such as water and methanol via hydrolysis/solvolysis of the lactone moieties. Therefore, stock solutions and working standards must be prepared in a non-nucleophilic solvent such as acetonitrile.] For obtaining a calibration curve drug-free plasma was spiked with picrotoxinin concentrations ranging from 25 nM to 10 μM. These samples were used immediately.

*Plasma sample preparation*: SPE cartridges (Hypersep C18, 100 mg, 1 mL, Thermo Scientific) were conditioned with 2 × 1 mL acetonitrile followed by 2 × 1 mL of water (Optima^®^, LC/MS grade, Fisher Chemical). After loading the SPE cartridges with plasma samples, they were washed with 1 mL of 20% acetonitrile in water followed by elution with 2 mL of acetonitrile. Eluted fractions were collected and evaporated to dryness, under a constant flow of air, using a PIERCE Reacti-Vap™ III evaporator (PIERCE, Il, USA). The residues were reconstituted using 200 μL acetonitrile and were used for LC–MS analysis.

*Brain/Liver sample preparation:* Approximately 200 mg of exactly weighed brain or liver tissues were added to 4.0 mL of acetonitrile and homogenized thoroughly using a T25 digital ULTRA-TURRAX^®^ homogenizer (IKA^®^ Works Inc., NC). The homogenized samples were centrifuged for 10 min at 8608×*g*, the supernatant separated and evaporated under a constant air flow as described above. The residues were reconstituted in 200 μL acetonitrile. The reconstituted material was loaded onto the preconditioned SPE cartridges and then eluted with 2 mL of acetonitrile. Load and elute fractions were collected and evaporated to dryness. The residues were reconstituted with 200 μL acetonitrile and were used for LC–MS analysis. [Please note that picrotoxinin degrades in tissue samples even when stored at − 80 °C and that samples, therefore, should be analyzed as quickly as possible. We noted that if the same brain samples were prepped again after 9 months, they showed a 50% lower picrotoxinin concentration on analysis.]

*LC/MS analysis:* LC/MS analysis was performed with a Waters Acquity UPLC (Waters, NY) equipped with an Acquity UPLC BEH 1.7 μm C-18 2.1 × 50 mm column, heated at 40 °C and interfaced to a TSQ Quantum Access Max mass spectrometer (Thermo Fisher Scientific, Waltham, MA). A gradient elution starting with 90% mobile phase A and 10% mobile phase B for 2 min was gradually taken to 25% mobile phase A and 75% mobile phase B at 2.10 min and then was kept at this ratio till 4.0 min. The gradient was then gradually taken back to 90% mobile phase A and 10% mobile phase B at 4.10 min. Total run time was 6 min using 10 mM ammonium formate solution in water as mobile phase A and acetonitrile as mobile phase B. The injection volume was 3.0 μL. Under these conditions, picrotoxinin had a retention time of 3.20 min. Using an Atmospheric Pressure Chemical Ionization (APCI) source in negative ion mode, capillary temperature 300 °C, vaporizer temperature 300 °C, discharge current 4.0 μA, sheath gas pressure (N_2_) 10 units, auxiliary gas pressure (N_2_) 5 units, picrotoxinin was analyzed by the selective reaction monitoring (SRM) transition of its negatively charged adduct with a formate ion at 337.04 (M + 45)^−^ into 203.01, and 109.14 *m/z*. A 9-point “plasma” calibration curve was developed for quantification. For this picrotoxinin was spiked into blank plasma at concentrations ranging from 25 nM to 10 μM (Supplementary Fig. 1). A “neat” calibration curve was run in parallel for assessment of the performance of the assay and for recovery. All calibration and analytical samples were injected in triplicate.

The HRMS experiments for the identification of the picrotoxinin hydrolysis product were performed on a Bruker TOF mass spectrometer (Bruker, San Jose, CA) in negative ion mode by direct infusion of 1 µM of picrotoxinin solution in pH 9.0 water.

### Liver microsome metabolism and plasma protein binding studies with TETS

A solution of 4 µL of [^14^C]-TETS in acetone at 152,000 cpm (counts per minute; 4.88 nmol) was placed in a glass tube and left to evaporate for 10 min. 95 µL of pooled mouse liver microsome solution at 1 mg/mL in PBS (lot 1310028, XenoTech LLC, Kansas City, KS) was added and the resulting mixture was incubated at 37 °C for 5 min before 5 µL of NADPH solution (20 mM) were added and the resulting mixture was incubated at 37 °C for 1.5 h. Two thin layer chromatography (TLC) plates were spotted with 6 µL of the resulting solution and were run in ethylacetate/hexane = 50:50 or 30:70 mixtures. The TLC plates were dried, wrapped in saran film together with a TLC plate with spotted standards (4–4000 cpm) and pressed against a phosphor screen (Molecular Dynamics, GE Amersham) for 2 days. The phosphor screen was then read on a 9400 Typhoon imager and is shown in Supplementary Fig. 3. The intense black spots correspond to [^14^C]-TETS. The nonmigrating spot at the bottom of the TLC is due to physically trapped TETS in the protein precipitate. The liver microsomes used in this experiment were active in control MROD (methoxyresorufin-*O*-deethylase) and EROD (ethoxyresorufin-*O*-deethylase) assays. Methoxyresorufin (MR) and ethoxysorufin (ER) are substrates for CYP1A enzymes and are turned into fluorescent resorufin. The rates of these model substrates were as predicted by the supplier. Details of the assay were previously described (Goswami et al. 2015).

TETS plasma protein binding was determined with mouse plasma using rapid equilibrium devices (RED, Fisher Scientific) according to the manufacturer’s protocol. [^14^C]-TETS at 1500 cpm was used per experiment. First, the time required to reach equilibrium for TETS was evaluated. Although the time to reach equilibrium was found to be less than 1 h, a 4 h incubation was used. The assay was run in pentaplicate.

### TETS pharmacokinetics

For intravenous application, TETS was diluted to 0.1 mg/mL in 90% saline and 10% DMSO. Adult male NIH-NCI Swiss mice (14–18 weeks of age, 18–25 g) were anesthetized with 1.5–2% isoflurane (Piramal Enterprises, Telangana India) in 100% oxygen at 1 L/min and maintained under isoflurane for the duration of the experiment to prevent seizures. TETS was administered intravenously at 0.05 mg/kg through the jugular vein and mice were randomized to draw blood at 3 of the following time points: 1 min, 5 min, 10 min, 20 min, 40 min, or 60 min after i.v. application. At the three time-points, 200 μL of blood was collected first from the left femoral vein, then the right femoral vein, and finally the vena cava before euthanasia (*n* = 3, 2 and 5, respectively). The collected blood was allowed to clot, serum collected, and stored at − 80 °C pending analysis. A second group of mice was dosed intraperitoneally with 0.2 mg/kg TETS (0.02 mg/mL in 90% saline and 10% DMSO) while being maintained under isoflurane anesthesia. Mice were euthanized at 5, 7.5 and 10 min post-TETS exposure by exsanguination via cardiac puncture (*n* = 3 per time point). Brains from the 10 min time point were used for electrophysiology.

For the long-term PK study, we utilized the so-called “TETS SE model”, in which adult male NIH-NCI Swiss mice are pretreated with riluzole 10 min prior to TETS administration (Zolkowska et al. 2018). Riluzole was prepared in 10% Captisol® (Ligand Technology, La Jolla, CA) and 90% saline at 10 mg/mL and further diluted 1:10 in sterile saline for i.p. injection at 10 mg/kg. TETS was prepared as described above and administered i.p. at 0.2 mg/kg. Mice were placed in individual plastic cages without bedding and seizure behavior was monitored. Midazolam (1.8 mg/kg) was administered intramuscularly at 40 min after the first myoclonic jerk to stop seizure activity and rescue the animals. Mice were anesthetized with 5% isoflurane and euthanized by exsanguination at 10, 30, 60 min, 2 h, 4 h, 8 h, 24 h, 48 h, 72 h, 7 days or 14 days post TETS exposure (*n* = 3 per time point). Brain, liver, kidneys and serum were collected and snap frozen for later analysis.

For the electrophysiological experiments shown in Fig. 4, mice were dosed using the TETS SE model as described above, and then euthanized at 2 and 14 days to collect serum. Serum collected from 5–7 mice per time point was pooled for the patch-clamp studies. We also collected brains from the animals euthanized on day 2 post exposure.

### TETS analysis by GC/MS and ELISA

TETS concentrations in mouse serum were initially analyzed using both GC/MS and an indirect competition ELISA to confirm our previous observation that the two assays render comparable results (Vasylieva et al. 2017). For the GC/MS assay 5 µL of 4 µg/mL [^13^C]-TETS were spiked into all serum samples (30 μL) as an internal standard. Samples were then vortexed vigorously for 1 min and 65 µL of methanol were added to the solution to precipitate the protein. The mixture was again vortexed for 1 min, left to stand for 5 min before centrifugation at 16,100 *g* for 10 min. The supernatant was then transferred to sample vials for the subsequent GC/MS analysis, which was performed as previously described (Vasylieva et al. 2017).

Serum samples were used directly for the ELISA. Matrix effects were accounted for using calibration curves contained 5% blank matrix (Vasylieva et al. 2017). Brain, liver and kidney samples required extraction before analysis. Method development for the extraction of TETS from tissue was performed using [^14^C]-TETS spiked tissue samples. After examining several solvents for liquid/liquid extraction, we decided to use solid-phase extraction (SPE) for an easier sample clean up. After finding that standard C8 and C18 columns were unable to retain [^14^C]-TETS, we investigated C18-OH, CBD and HLB columns. Waters Oasis PRIME HLB columns (Waters, Milford, MA) were best able to retain [^14^C]-TETS during two wash steps and before elution with methanol (98%). For sample preparation, each brain, liver or kidney sample was weighed, placed in a 1.5 mL Eppendorf tube, and homogenized in 1 mL of a 20% methanol/water mixture using a SPEX 1600 MiniG Automated Tissue Homogenizer and Cell Lyser (SPEX, Metuchen, NJ). To separate tissue from the supernatant, tubes were spun for 10 min at 16,873 × g. The supernatant was then added to pre-conditioned HLB columns and the Eppendorf tubes were discarded. The samples on the HLB columns were washed with 2 mL of water, 2 mL of 40% methanol/water and finally eluted with 3 mL of 98% methanol. Samples were evaporated using a SpeedVac Vacuum and reconstituted for the ELISA assay.

### Electrophysiological recordings on HEK cells expressing the α_2_β_3_γ_2L_ GABA_A_ receptor

The human GABA_A_ receptors α_2_, β_3_, and γ_2L_ cloned into pcDNA3.1 expression vectors were a gift from Dr. Robert L. Macdonald, Vanderbilt University, TN. L929 cells, a mouse fibroblast cell line (CCL-1), were obtained from ATCC (American Type Culture Collection, Manassas, VA, United States) and cultured in Dulbecco's modified Eagle's medium (Lonza) supplemented with 10% fetal bovine serum, 100 U/mL penicillin and 100 mg/mL streptomycin (Invitrogen) and maintained in humidified 95% air and 5% CO_2_ at 37 °C. L929 cells were transfected using FuGENE 6 (Fisher Scientific) transfection reagent in Opti-MEM® reduced serum medium (Life Technologies, Benicia, CA) with an equal amount of each of the subunits (1:1:1) in combination with pEGFP-C1 (Invitrogen). The ratio of total cDNA to transfection reagent was 2:1. Cells were detached by trypsinization 48 h post-transfection, washed, and plated onto poly-L-lysine coated glass cover-slips. Transfected cells were identified using an epifluorescence microscope.

Whole-cell voltage-clamp recordings were performed at room temperature with an EPC-10 HEKA amplifier. Cells were bathed in an external Ringer solution consisting of 160 mM NaCl, 4.5 mM KCl, 1 mM MgCl_2_, 2 mM CaCl_2_, 10 mM HEPES, pH 7.4, 308 mOsm. Recording electrodes were pulled from soda-lime glass micro-hematocrit tubes (Kimble Chase, Rochester, NY) and fire-polished to resistances of 1.8–3 MΩ. Electrodes were filled with an internal solution consisting of 154 mM KCl, 2 mM CaCl_2_, 1 mM MgCl_2_, 10 mM HEPES and 10 mM EGTA, pH 7.2 and 302 mOsm. Cells were voltage clamped at -80 mV and control currents were recorded under the application of EC_90_ GABA using a gravity-fed fast perfusion system (VC38 system, ALA Scientific), for 5 s followed by a 50-s wash with Ringer solution. Pooled serum samples or brain samples were loaded onto solid-phase extraction columns. TETS was then eluted with methanol as described for the ELISA assay. The eluent was evaporated to dryness. Samples were then reconstituted with Ringers at the original loading volume and were vortexed. Extracts were perfused onto cells after eliciting control currents and allowed to sit for 3 min on the cell before application of EC_90_ GABA. For experiments with pooled, reconstituted serum, data are the average current inhibition from 4–9 cells per time point.

## Results

### Picrotoxinin is unstable in aqueous solutions

Since picrotoxinin contains two lactone moieties (Fig. 1a) that can potentially be opened in nucleophilic solvents, we more closely investigate the stability of picrotoxinin in aqueous solutions at acidic pH (4.0), at physiological pH (7.4) and alkaline pH (12.0) at both room temperature and at 37 °C. Since we could only find a 30-year HPLC method in the literature (Soto-Otero et al. 1989) we first developed a new LC/MS method for picrotoxinin. Picrotoxinin is a member of a highly oxygenated group of sesquiterpenes called picrotoxanes, which, in general, have very poor ionization efficiency in both heated electrospray ionization (HESI) or atmospheric-pressure chemical ionization (APCI) mode. We also could not obtain a quasi-molecular ion of picrotoxinin in positive or negative mode with the HESI source, while APCI in negative-polarity mode furnished a quasi-molecular ion in very low abundance at *m/z* = 291.1 (M−1)^−^, which was unusable for method development. On the other hand, we observed that picrotoxinin readily forms adducts with negatively charged ions such as formate, *m/z* = 337.04 (M + 45)^−^, which, in turn, produced two prominent product ions of *m/z* = 203.0 and 109.1 (Supplementary Fig. 2A). To confirm the identity of the adduct, we changed the counterion from formate to acetate and found the acetate adduct, *m/z* = 351.04 (M + 59)^−^, to produce the same product ions of *m/z* = 203.0 and 109.1 (Supplementary Fig. 2B). We also studied the MS/MS of picrotoxinin’s low abundant quasi molecular ion *m/z* = 291.1 (M−1)^−^ and found the same product ions of *m/z* = 203.0 and 109.1 (Supplementary Fig. 2C). Therefore, a method based on the fragmentation of picrotoxinin’s most abundant formate adduct into product ions was developed for this study. A literature survey revealed that Larsen et al. also relied on the formate adduct of other picrotoxanes namely tutin, dihydrotutin, hyenanchin (mellitoxin) and dihydrohyenanchin to develop LC/MS methods for the analysis of these toxins in honey (Larsen et al. 2015). Using this newly developed LC/MS assay to follow picrotoxinin stability over time we found that picrotoxinin degrades instantaneously at pH 12.0 (Fig. 1b). At physiological pH roughly 70% of picrotoxinin degrades within one hour at both room temperature and 37 °C, while it is more stable at an acidic pH of 4.0, where 75% remain intact after 24 h. TETS, in contrast, was stable at acidic, neutral and alkaline pH (not shown), thus confirming its previously published long-term stability in drinking water (Knaack et al. 2014).

### Picrotoxinin has a plasma protein binding of 50% and rapidly degrades in plasma to picrotoxic acid

**Fig. 2:**
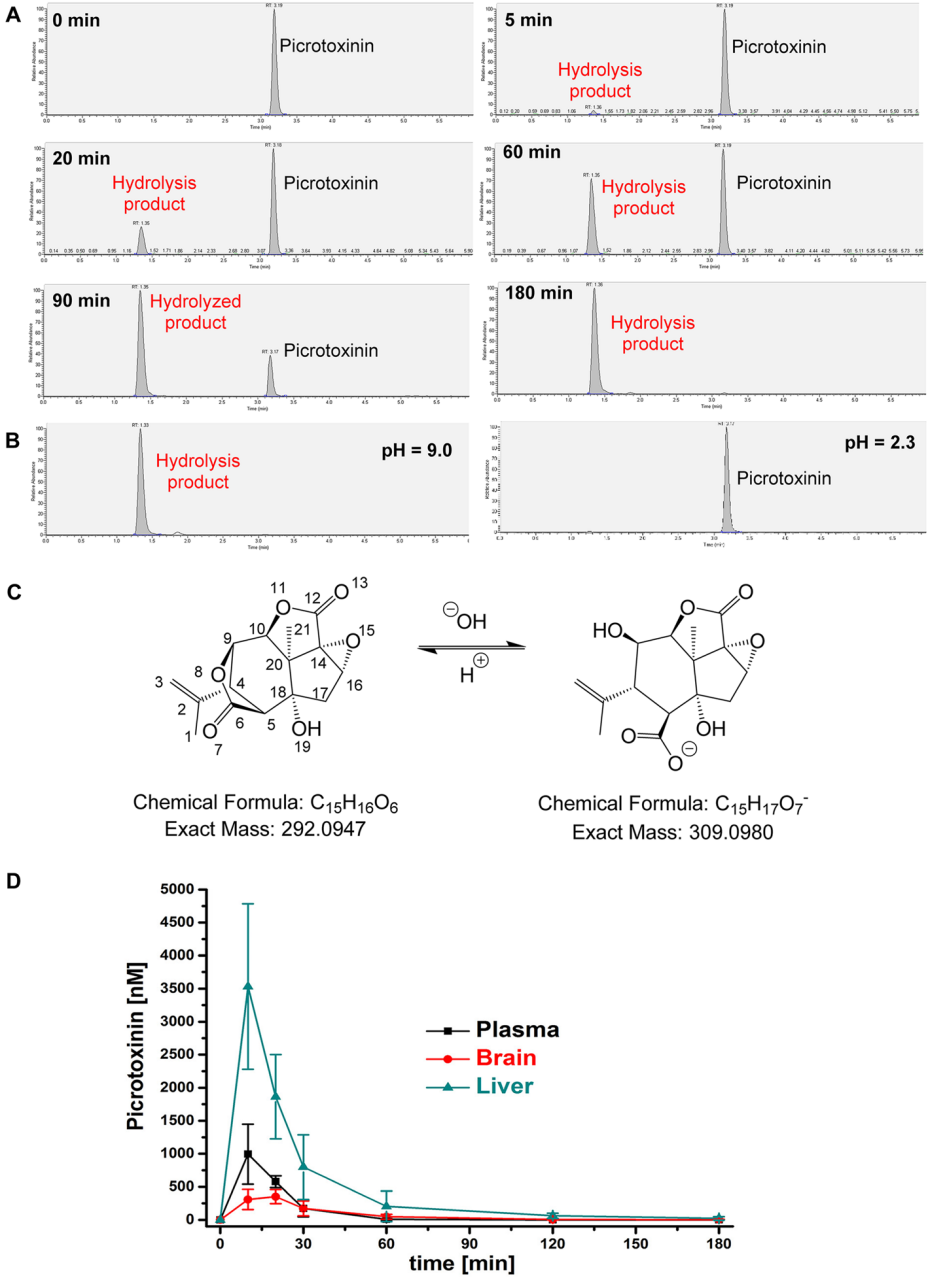
Picrotoxinin hydrolyses in plasma and has a short in vivo half-life. **a** LC/MS chromatograms of picrotoxinin after 0, 5, 20, 60, 90 and 180 min of incubation in mouse plasma. Picrotoxinin has a retention time of 3.19 min. The hydrolysis product has a retention time of 1.35 min. **b** Incubation of picrotoxinin at pH 9 produces the same hydrolysis product, while acidification can convert the hydrolysis product back into picrotoxinin. **c** Suggested structure of the hydrolysis product based on high-resolution mass spectrometry. **d** Total picrotoxinin plasma, liver and brain concentrations in mice; *n* = 6–9 mice per time point; shown mean ± SD values in nM

We next determined picrotoxinin’s plasma protein binding. In the absence of plasma protein, picrotoxinin required 1 h to reach equilibrium distribution in a rapid equilibrium device (not shown). However, when the same experiment was repeated in mouse plasma, picrotoxinin concentrations declined in both chambers before equilibrium was reached. We subsequently studied the stability of picrotoxinin in mouse plasma using LC/MS. Within 5 min, a new peak with an earlier retention time (1.35 min) than picrotoxinin (3.17 min) appeared in the LC chromatogram (Fig. 2a). While this peak was initially small, it rapidly grew until all picrotoxinin had vanished at 180 min (Fig. 2a). The same hydrolyzed product peak appeared when picrotoxinin was incubated in water at pH 9.0 (Fig. 2b), but could be converted back into picrotoxinin by addition of formic acid (0.1% V/V, pH 2.3), suggesting that one, or both lactone rings of picrotoxinin, are being opened and closed in a pH dependent manner.

To identify the hydrolysis product we performed high-resolution mass spectroscopic (HRMS) analysis on a Bruker TOF (Time of Flight) mass spectrometer. As shown in Supplementary Fig. 3, a strong signal at *m/z* of 309.0979 was found in negative ion mode, indicating the presence of a product with the molecular formula C_15_H_17_O_7_ resulting from the hydrolysis of only one of the two lactone moieties of picrotoxinin (HRMS (ESI): *m/z* calculated for C_15_H_17_O_7_ (M−H)^−^: 309.0980; found: 309.0979). This finding, of course, raised the question of which one of the two lactone rings is being hydrolyzed? The bridged bicyclic lactone ring is less sterically hindered and, therefore, has a higher chance of a nucleophilic attack on carbonyl carbon number 6 by a hydroxide ion resulting in picrotoxic acid (Fig. 2c) as the hydrolysis product (for structural confirmation see Supplementary Fig. 4).

Since these experiments demonstrated that picrotoxinin hydrolyzes rapidly in plasma, presumably both chemically and catalyzed by plasma esterases, we decided that it was not feasible to determine plasma protein binding by equilibrium dialysis. We instead used ultracentrifugation with a short 10 min spin and found a plasma protein binding of 54.6 ± 11.2% (*n* = 3) with this method.

### Picrotoxinin is rapidly metabolized in vivo

Adult male NIH-NCI Swiss mice were dosed with 2 mg/kg picrotoxinin and total picrotoxinin concentrations in plasma, brain and liver determined at multiple time points. As shown in Fig. 2d, picrotoxinin has a short half-life (*t*_1/2_ ~ 15 min) and penetrates moderately well into the brain. While total liver concentrations were ~ threefold higher than plasma concentrations, brain concentrations were lower than plasma concentrations and peaked at 351 ± 106 nM at 20 min and then rapidly fell below the detection limit at 2 h. These concentrations, which would block roughly 10–20% of GABA_A_ current based on picrotoxinin’s IC_50_ in electrophysiological experiments (Pressly et al. 2018), were, however, sufficient to induce seizures, since 75% of the 42 mice in our study exhibited seizures (score 3–5 on the Racine scale) and 25% died during the experiments. Death typically occurred between 20 and 30 min after picrotoxinin administration and brain concentrations in these animals were found to be 376 ± 120 nM (*n* = 10).

### TETS is metabolically stable and persists in vivo

Before performing in vivo studies with TETS, we determined its metabolic stability and plasma protein binding. Incubation of [^14^C]-TETS with mouse liver microsomes demonstrated that TETS is not metabolized (Supplementary Fig. 5). Using equilibrium dialysis we found that TETS has a relatively low plasma protein binding of only 4.7 ± 2.9% (*n* = 5).

**Fig. 3:**
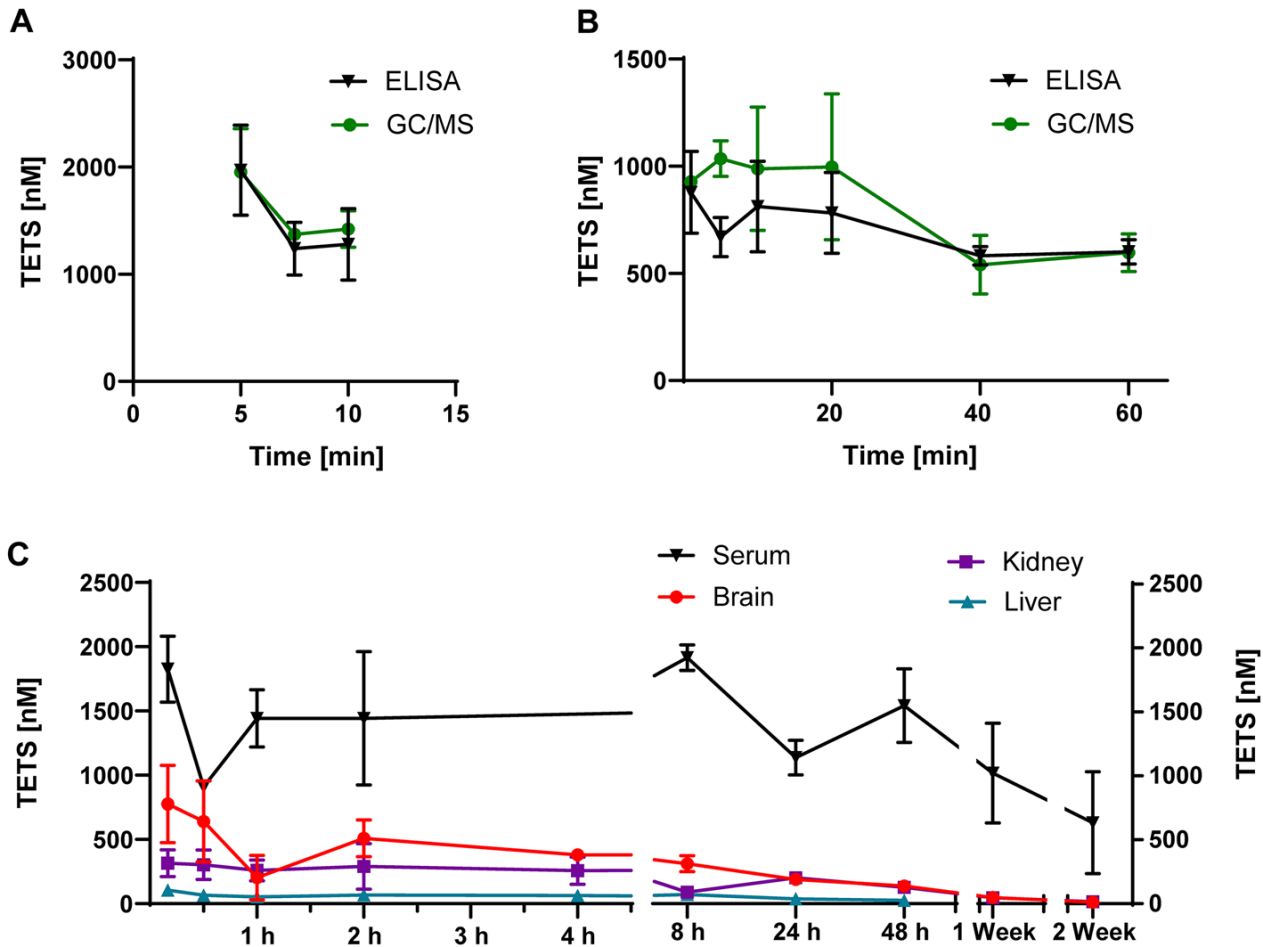
TETS persist in vivo. **a** Pilot experiment comparing the sensitivity of the TETS ELISA to GC/MS following i.p. administration of 0.2 m/kg TETS to mice (*n* = 3). Shown are serum concentration as mean ± SD. **b** TETS serum concentration following i.v. bolus administration of 0.05 mg/kg TETS (*n* = 5). **c** Long-term pharmacokinetic experiment showing TETS concentrations in serum, brain, kidney and liver over a time course of 2 weeks (*n* = 3 mice per time point). Shown are total concentrations as mean ± SD

We next wanted to study the pharmacokinetics of TETS but were concerned about whether the detection limits of the available analytical assays would allow us to follow TETS concentrations over time. We, therefore, started with a short pilot experiment in which we administered TETS intraperitoneally at 0.2 mg/kg to adult male NIH Swiss mice anesthetized with isoflurane to prevent seizures and took blood at 5 min, 7.5 min and 10 min. These blood samples were used to compare our two analytical methods, GC/MS (Zhao et al. 2014) and an indirect competition ELISA our group developed (Vasylieva et al. 2017). The two assays produced similar results as shown in Fig. 3a. Both assays showed that administration of 0.2 mg/kg of TETS resulted in serum concentrations of 1.973 ± 0.42 μM at 5 min, 1.236 ± 0.245 μM at 7.5 min and 1.277 ± 0.333 μM at 10 min as detected by ELISA, with a 94% correlation with the GC/MS method. This pilot study showed that we were well within the detection range of both the GC/MS and the ELISA assay. Since the ELISA assay has a much better limit of detection (1 ng/mL with mouse serum) than the 50 ng/mL for the GC/MS assay (Vasylieva et al. 2017), we decided to use the ELISA assay.

We next administered a lower dose of 0.05 mg/kg TETS by intravenous bolus to determine standard pharmacokinetic parameters. To our surprise, we observed that TETS serum concentrations basically “flat-lined” at ~ 600 nM in blood samples taken at 1 min, 5 min, 10 min, 20 min, 40 min, 60 min instead of decreasing with the normally observed mono- or biexponential decay. To further investigate this persistence, we next designed a long-term PK experiment in the “TETS SE model”, in which adult male NIH-NCI Swiss mice are pretreated with riluzole 10 min prior to TETS administration (Zolkowska et al. 2018). The riluzole pretreatment prevents mice from succumbing to tonic hindlimb extension, and thus the majority of animals go into a prolonged *status epilepticus* following i.p. administration of 0.2 mg/kg TETS. To prevent death from TETS-induced *status epilepticus*, seizures are terminated by intramuscular administration of midazolam 40 min after the first myoclonic jerk. Mice were euthanized at 10 min, 30 min, 60 min, 2 h, 4 h, 8 h, 24 h, 48 h, 72 h, 7 days or 14 days post-TETS exposure and TETS serum, brain, liver, and kidney concentrations were analyzed using the TETS selective immuno-assay. As shown in Fig. 3d, TETS serum concentrations remained around 400 ng/mL (= 1.6 μM) for 48 h and then slowly started to fall between 7 and 14 days.

**Fig. 4:**
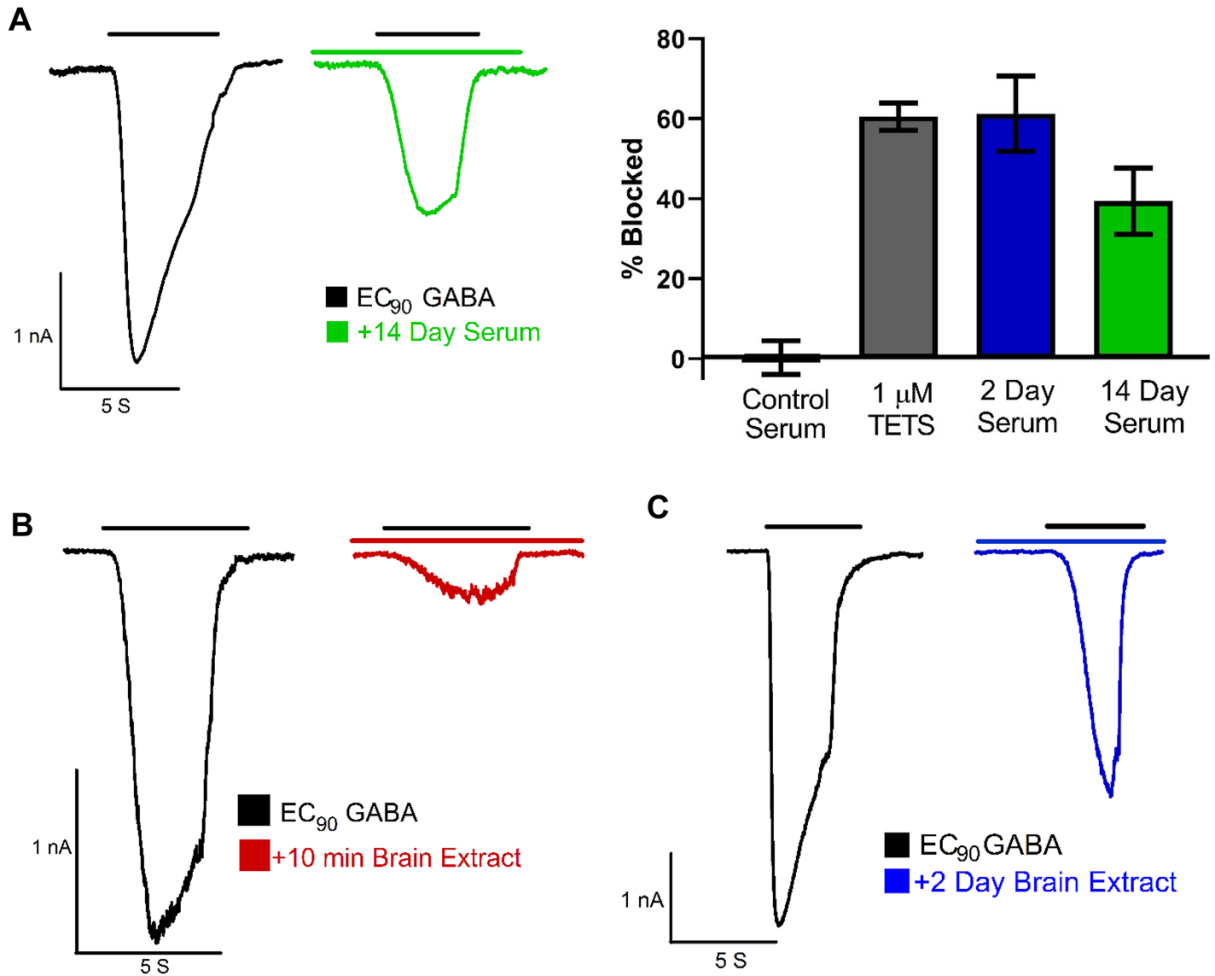
TETS remains pharmacodynamically active in vivo for 14 days. **a** Pooled serum from mice sacrificed 2 days or 14 days after administration of 0.2 mg/kg TETS blocks current through recombinantly expressed α_2_β_3_γ_2_ GABA_A_ receptors. Control currents were elicited by 40 μM GABA, which corresponds to the EC_90_ for this receptor combination. After washout, serum extracts were perfused and currents again elicited by 40 μM GABA. Only recordings where TETS could subsequently be washed out were used for quantification of the blocking effect. Control serum: 1.3 ± 4.2% (*n* = 5 cells); 1 μM TETS: 60.5 ± 4.4% current block (*n* = 8 cells); pooled 2-day serum: 61.3 ± 9.4% current block (*n* = 4 cells); pooled 14-day serum: 39.4 ± 8.3% current block (*n* = 7 cells). Data are mean ± SD. Inhibition of GABA currents by extracts from mouse brain removed 10 min (**b**) and 2 days (**c**) after TETS administration

We next developed extraction methods using [^14^C]-TETS as a reference standard to use the ELISA to measure TETS concentrations in tissues. In keeping with its relatively high polarity, TETS concentrations in brain and kidney were lower than in serum but showed a similar persistence over time (Fig. 4d). Interestingly, TETS concentrations in the liver were very low, about 10–20-fold lower than serum, and dropped below the detection limit after 48 h. TETS clearance from tissues began at 8 h post-exposure (Fig. 3d).

### TETS remains pharmacodynamically active in vivo for 14 days

To determine whether the TETS detected by the ELISA is pharmacodynamically active, we pooled serum from several mice that had been sacrificed on day 2 (*n* = 5) or day 14 (*n* = 7), cleaned it up as described for the ELISA assay and then reconstituted with Ringers solution. This solution was then perfused onto L929 cells that were transiently transfected with α_2_β_3_γ_2_ GABA_A_ receptors, the receptor combination that is most sensitive to TETS inhibition (Pressly et al. 2018). As shown in Fig. 4a pooled serum taken from mice on day 2 blocked currents elicited by EC_90_ GABA by 61.3 ± 9.4%, comparable to the block produced by 1 μM of TETS in the same experiment (60.5 ± 4.4%). Pooled serum taken from mice 14 days after TETS administration still blocked GABA-induced currents by roughly 40% (39.4 ± 8.2%), confirming the long-term TETS persistence we had detected by ELISA. Control serum subjected to the same purification process had no GABA_A_ receptor activating or inhibiting activity (1.3 ± 4.2%). We also extracted two brain samples removed 10 min or 2 days after TETS administration and found that the extracts from these samples blocked GABA induced currents by ~ 85% (Fig. 4b) and by ~ 50% (Fig. 4c), respectively, after reconstitution in Ringers solution. Taken together, these results confirm that TETS maintains its GABA_A_ receptor blocking activity in vivo for at least 14 days.

## Discussion

The United States Department of Homeland Security lists both the proconvulsant GABA_A_ receptor antagonists picrotoxin and TETS as credible threat agents. Of these two compounds, TETS is by far the more serious threat agent because of its extreme toxicity and its ease of synthesis (Laukova et al. 2019b). In contrast to the tasteless and odorless TETS, which is easy to administer in food or drink, picrotoxin is extremely bitter making it unlikely for any animal or human to voluntarily consume toxic; let alone lethal doses. If used with malicious intent or as a pharmacological tool to induce seizures in experimental animals, picrotoxin or its active component, picrotoxinin, therefore, need to be administered parenterally. Picrotoxinin is sensitive to hydrolysis (Fig. 1) and has a very short in vivo half-life (Fig. 2). These characteristics, combined with the difficulty of obtaining large quantities of this natural product, make it highly unlikely for picrotoxin to ever be used as a chemical weapon. The rodenticide TETS, in contrast, constitutes a chemical agent of serious concern. As previously reported (Knaack et al. 2014), it is stable in drinking water for months. We now demonstrate here that TETS is not subject to metabolism by liver microsomes and that it persists in the body following acute intoxication. Mice administered TETS at the LD_50_ of 0.2 mg/kg in the presence of rescue pre- and post-medications to enable them to survive prolonged *status epilepticus*, exhibited serum levels that remained constant at around 1.6 μM for 48 h before they started to slowly fall (Fig. 3d). Tissues like brain, kidney and liver showed a similar persistence, yet at lower levels in keeping with the polarity of TETS. Of these three tissues, brain showed the highest TETS concentrations, and, although TETS does not enrich in the brain based on our findings, it is relatively well brain penetrant with a brain/serum ratio of ~ 0.3. Brain concentrations peaked at 10 min at 200 ng/g, which equals 0.8 μM, a concentration that considering the low protein binding of TETS, should be sufficient to block ~ 65% of GABA_A_ current carried by α_2_β_3_γ_2_ receptors and 15–20% of current through other GABA_A_ receptors. Brain concentrations subsequently persisted above 400 nM for the next 8 h before TETS started to slowly clear from the brain over the next 7 days (Fig. 3).

This persistence of TETS in both serum and brain tissue might explain the prolonged seizures observed in humans following TETS exposure (Laukova et al. 2019b). For example, in a well-documented case occurring in the United States, a 15-month old girl found to be playing with illegally imported TETS from China exhibited seizures lasting 4 days and subsequently developed epilepsy and remained mentally impaired (Barrueto et al. 2003). A retrospective study of 370 patients who survived TETS poisoning in China, documented that 55% of patients developed epilepsy despite receiving antiseizure medications following the initial exposure, and even several patients who did not report having experienced clinically detectable seizures following the TETS exposure developed epilepsy (Deng et al. 2012). Similarly, a 9-year old, female mixed-breed dog, that was seen in the Peace Avenue Veterinary clinic in Hong Kong, exhibit neurological symptoms and seizures for at least 6–7 days after a suspected TETS ingestion, which was confirmed when the treating veterinarian shipped a urine sample collected on the second day of hospitalization to UC Davis where we found it to contain 179 ng/mL (= 745 nM) TETS using our TETS-selective immunoassay. [The clinical details of this case will be published in a separate veterinary case report.] Clinical observations in both humans and companion animals thus are consistent with a sustained depression of GABA_A_ signalling following TETS exposure. In contrast to humans, who clearly exhibit prolonged seizure activity in response to TETS, we suspect that the mouse brain must evoke some compensatory plasticity mechanisms that counteract the persistent GABA_A_ receptor inhibition because they do not continue to seize despite the continuing presence of TETS, we demonstrated here. Interestingly, a previous study exploring whether mice could be “kindled” by repeated low dose administrations of TETS found that repeated administrations of 0.05 mg/kg TETS, while resulting in a transient increase in seizure score on the second and third treatment, did no longer elevate seizure severity at subsequent administrations (Zolkowska et al. 2012). This was interpreted by the authors of this study as TETS being different from other GABA_A_ antagonists like PTZ, which are able to increase seizure susceptibility in rodents with repeated administration. We speculate that these differences between humans and rodents are probably due to a greater ability of the mouse brain to compensate for the inhibition of the TETS sensitive GABA_A_ subtypes by either increasing the expression of less TETS sensitive subtypes or other mechanisms that reduce neuronal excitability.

While picrotoxin is not likely to be used as a threat agent in a real-world scenario, our study can provide some guidance for the use of both picrotoxin and picrotoxinin as pharmacological tool compounds. Picrotoxin is only sparingly soluble in water and even the Merck index in its 10th and current online version suggests making picrotoxin more soluble by alkalizing the solution with ammonia or NaOH (https://www.rsc.org/Merck-Index/monograph/m8797/picrotoxin). We here demonstrate that a significant amount of picrotoxinin hydrolyses at pH 7.4 (75%) within 1 h of preparing solutions and that hydrolysis is even faster at more alkaline pH values (Fig. 1b). We, therefore, suggest that the large variations in the seizure-inducing and lethal doses of picrotoxin reported in the literature (Cymerblit-Sabba and Schiller 2010; Klaassen et al. 2006; Makinson et al. 2014; Shimshoni et al. 2008) are caused by variations in the time different laboratories take from preparing picrotoxin solutions to dosing animals. We recommend that picrotoxinin solutions always be freshly prepared immediately before dosing animals. Interestingly, clinicians using picrotoxinin in the 1940s to treat barbiturate overdosage were aware of its instability and short duration of action and used to administer 5 mg of picrotoxin every 15 min until the patient regained consciousness (Misir 1946). In addition to being sensitive to hydrolysis, picrotoxinin also has a very short in vivo half-live (~ 15 min in our experiments). Picrotoxinin penetrates into the brain moderately well with a brain/plasma ratio of ~ 0.3, but unlike TETS, is cleared from the brain very quickly as plasma levels fall (Fig. 2d). Interestingly, if picrotoxinin is administered by the intracerebroventricular route in a solvent where it is stable like DMSO, its toxicity is much higher and roughly similar to TETS with an LD_50_ of 0.1 mg/kg (Bloomquist 1992; Zolkowska et al. 2012).

The most significant and concerning the finding of our study is the long-term persistence of TETS in both the vascular compartment and in the brain following intoxication. Previous literature regarding this point has been contradictory. A radiotracer study performed in the 1970s had reported rapid excretion in mice and snowshoe hares (Radwan 1970), while a more recent study performed in rabbits reported a plasma half-life of 262 h following oral administration (Zhang et al. 2005). Using a TETS specific immunoassay with a low limit of detection (Vasylieva et al. 2017) and electrophysiology as a pharmacodynamic test of biological activity, we here demonstrate that TETS does persist as reported by Zhang et al*.* using gas chromatography with nitrogen phosphorus detection (Zhang et al. 2005). However, using our TETS-selective immunoassay we here demonstrated a tenfold lower detection limit in serum (LOD 1 ng/mL) than the nitrogen phosphorus detector (LOD 10 ng/mL), and, more importantly, also were able to analyze TETS concentrations in tissues.

We are somewhat puzzled to observe such persistence of TETS, which is more common with highly lipophilic compounds like dioxins or polychlorinated biphenyls (PCBs). TETS is a relatively polar compound that appears too polar and too sterically hindered to be rapidly metabolized by cytochrome P450 enzymes involved in xenobiotic metabolism. TETS has a very low plasma protein binding (5%) and, therefore, would be expected to be freely filtered in the kidney and to be as rapidly excreted as, for example, metformin, a diabetes drug that has no plasma protein binding (Pentikainen et al. 1979). The fact that TETS persists at a serum concentration of 1.6 μM for 48 h following intraperitoneal administration at 0.2 mg/kg suggests that TETS rapidly distributes into total body water and then escapes renal elimination through reabsorption by one or possibly multiple transporters. The bioavailability is excellent and likely to be close to 100% based on the fact that seizure severity and 1-h lethality of TETS is virtually the same following intraperitoneal, subcutaneous or oral administration and only somewhat delayed or reduced by oral administration in peanut butter, which is delaying absorption by prolonging the time for passage from the stomach to the small intestines but not reducing it (Laukova et al. 2019a).

In conclusion, we here show that the differences in toxicity between the two GABA_A_ antagonists picrotoxin and TETS are due to striking differences in pharmacokinetics and chemical properties. While picrotoxin is highly sensitive to hydrolysis and has a very short in vivo half-life, TETS is chemically and metabolically stable and shows long-term persistence in vivo*.* These differences have implications for the treatment of intoxications with these agents. Picrotoxin intoxications will only require short-term seizure control, while patients exposed to TETS require long-term monitoring to assure that seizures do not return. Indeed, more than 50% of patients who survived TETS poisoning in China, have reportedly developed epilepsy despite receiving antiseizure medications following the initial exposure (Deng et al. 2012). We, therefore, suggest that while seizure control is, of course, a life-saving countermeasure for TETS intoxication, it should ideally be combined with treatments that can accelerate the elimination of TETS, perhaps by preventing renal reabsorption (if that is the mechanism of the TETS persistence). This approach will require the future identification of the responsible transporters and, if no inhibitors are available, the development of inhibitors for these transporters. An alternative approach might be to design a TETS binding protein or develop a high-affinity anti-TETS nanobody to neutralize and eliminate TETS. In the absence of such specific countermeasures, the only effective means to remove TETS from the body following exposure remains extracorporeal blood purification, which ideally should be started early and be combined with hemoperfusion (HP) to first quickly lower TETS plasma levels with prolonged continuous venovenous hemofiltration (CVVH) to prevent rebound of TETS levels. This intervention has been shown to improve survival and reduce long-term consequences such as mental deficiency in a hospital in Nanjing that was treating 270 patients admitted after a TETS poisoning attack in 2002 (Dehua et al. 2006).

## Electronic supplementary material

Below is the link to the electronic supplementary material.

Supplementary file1 (PDF 663 kb)

## Abbreviations

cpm: Counts per minute
DMSO: Dimethylsulfoxide
ELISA: Enzyme-linked immunosorbent assay
GABA: Gamma-aminobutyric acid
GC/MS: Gas–liquid chromatography/mass spectrometry
LD_50_: Dose that is lethal in 50% of subjects
LC/MS: Liquid chromatography/mass spectrometry
HPLC: High pressure liquid chromatography
UPLC: Ultra high-pressure liquid chromatography
SD: Standard deviation
TETS: Tetramethylenedisulfotetramine
