# Supplementary material for: Comparison of the toxicokinetics of the convulsants picrotoxinin and tetramethylenedisulfotetramine (TETS) in mice
Source: Arch Toxicol. 2020 Apr 1;94(6):1995–2007. doi: 10.1007/s00204-020-02728-z (PMC7303059; doi:10.1007/s00204-020-02728-z)
Supplement: Supplementary file 1 — Supplementary file1 (PDF 663 kb) [file 204_2020_2728_MOESM1_ESM.pdf]

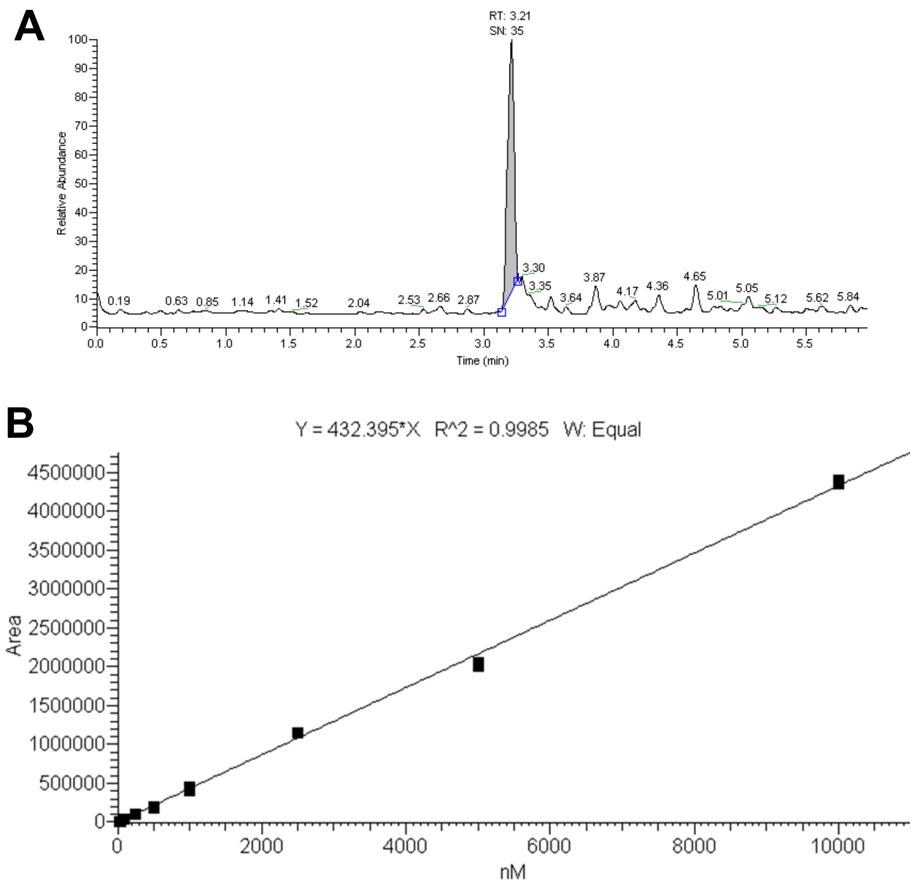

Picrotoxinin LC/MS assay. A) Chromatogram showing 25 nM of picrotoxinin in rat plasma. **B)** Calibration curve of picrotoxinin in rat plasma. The limit of detection for the assay is 5 nM. The limit of quantification is 15 nM.

Supplementary Figure 1

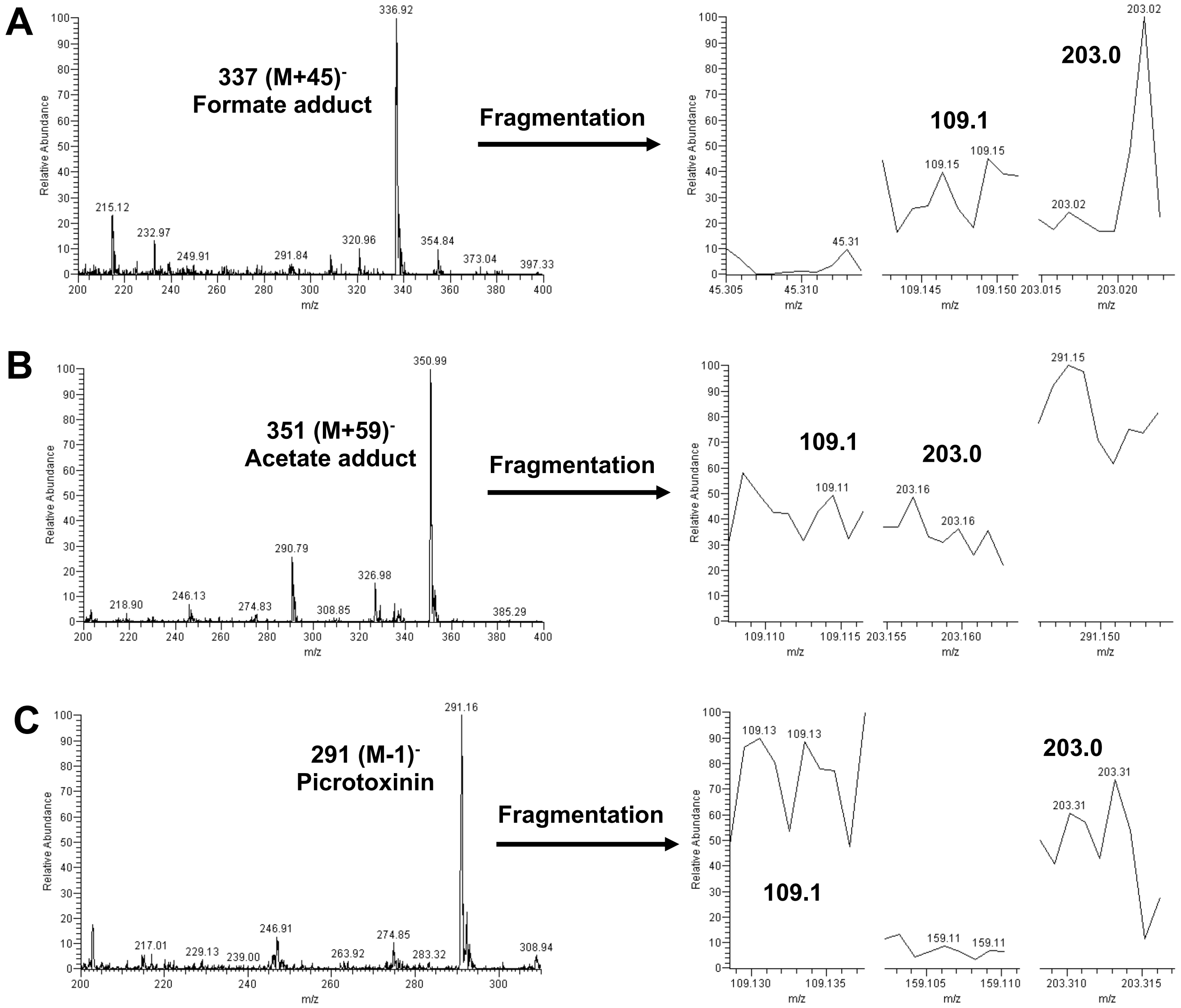

Ionization of picrotoxinin and its adducts using APCI in negative-polarity mode.
A) *Left,* Mass spectrum of picrotoxinin's negatively charged formate adduct $[(M+45)^{-} = 337$ m/z] on the first quadrupole of the mass spectrometer. *Right,* Fragmentation of the 337 adduct ion into its three most abundant product ions on the 3rd quadrupole of the mass spectrometer. **B)** *Left,* Mass spectrum of picrotoxinin's negatively charged acetate adduct $[(M+59)^{-} = 351$ m/z] on the first quadrupole of the mass spectrometer. *Right,* Fragmentation of the 351 adduct ion into its three most abundant product ions on the 3rd quadrupole of the mass spectrometer. **C)** *Left,* Mass spectrum of picrotoxinin's negatively charged quasi molecular ion $[(M-1)^{-} = 291$ m/z] on in the first quadrupole of the mass spectrometer. Right, Fragmentation of the 291 ion into its three most abundant product ions on the 3rd quadrupole of the mass spectrometer.

Supplementary Figure 2

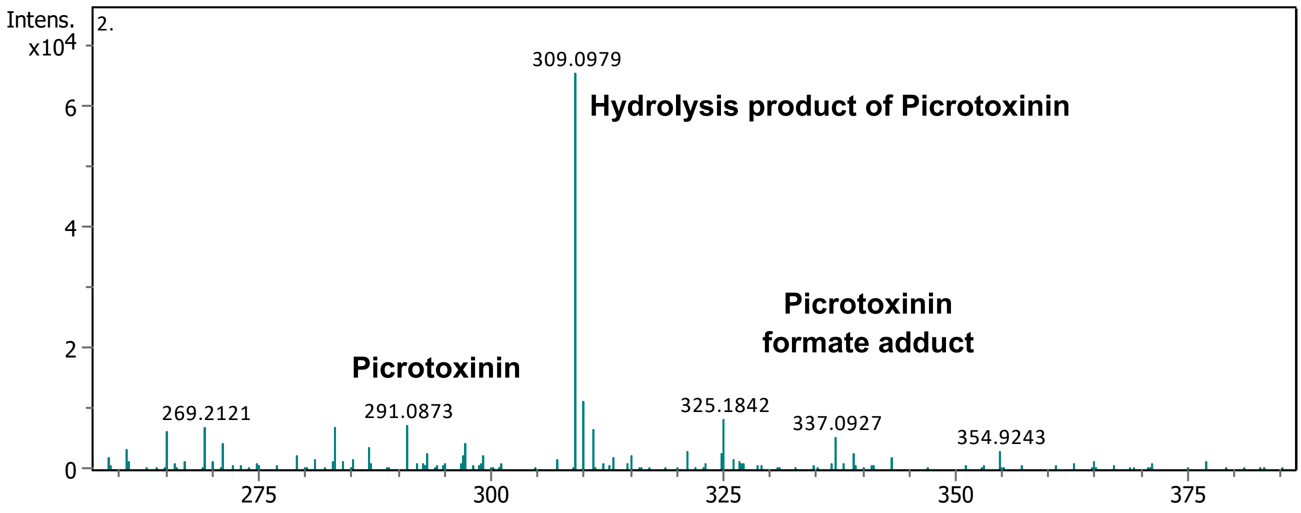

Spectrum Data

Meas. m/z	#	Ion Formula	m/z	err [ppm]	mSigma	# mSigma	Score
309.0979	1	$C_{15}H_{17}O_7$	309.0980	0.2	4.9	1	100.00
	2	$C_{12}H_9N_{10}O$	309.0966	-4.1	8.6	2	50.37
	3	$C_{16}H_{13}N_4O_3$	309.0993	4.6	9.2	3	45.89

Hydrolysis product of picrotoxinin. ***Top,*** High resolution mass spectrum of picrotoxinin in pH 9.0 water. The spectrum in the negative ion mode shows a small molecular ion peak for picrotoxinin at *m/z* of 291.0873 and a strong signal at *m/z* of 309.0979, which closely matches the molecular formula $C_{15}H_{17}O_7$ (blue highlight), suggesting the opening of one of the two picrotoxinin lactone rings. HRMS (ESI): *m/z* calcd. for $C_{15}H_{17}O_7$ $(M-H)^-$: 309.0980; found: 309.0979.

Supplementary Figure 3

Supplementary Figure 4

Pictrotoxinin hydrolyzes to picrotoxic acid. In order to investigate the mechanism and site of hydrolysis, we treated picrotoxinin with sodium methoxide in methanol and determined the structure of the product by single crystal X-ray diffraction which revealed that methyl picrotoxate (**A**) was formed as a result of the cleavage of the bridged bicyclic lactone ring by a methoxide anion, followed by intramolecular reaction of the newly formed alkoxide anion with the epoxide moiety of picrotoxinin, leading to the opening of the epoxide and formation of a new six membered ring in a tandem manner (**B**). Methyl picrotoxate is also a naturally occurring sesquiterpene (Krische et al. 1998).

A

Molecular structure of methyl picrotoxate determined by single crystal X-ray diffraction.
CCDC Number: 1982743

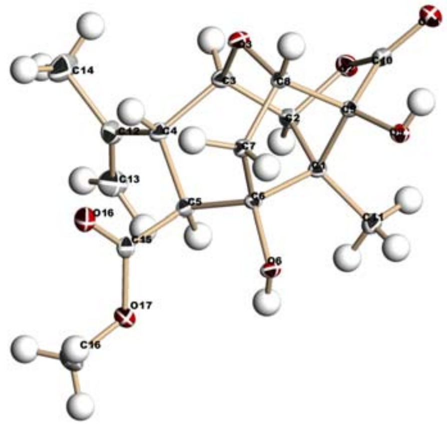

B

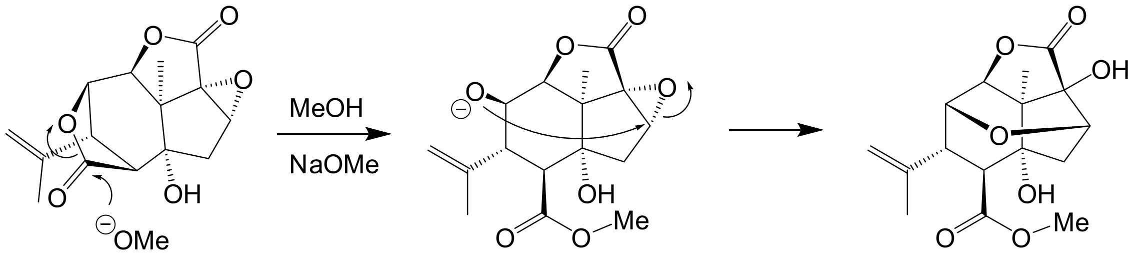

Methyl picrotoxate

While these results unambiguously determined the site of hydrolysis, we still had to answer the question of whether the hydrolysis of picrotoxinin would stop at the lactone ring opening step to furnish picrotoxic acid or would proceed to the rearranged product under mildly basic/neutral conditions (**C**). The fact that picrotoxinin hydrolysis is reversible in a pH dependent manner (Figure 2B), suggests that picrotoxic acid is the possible product as it is chemically feasible for the lactone ring to reversible open and close with changing pH. Also, in the presence of water and a weaker base, lactone ring opening would lead to the formation of an alcohol, rather than an alkoxide ion, which is not strong enough to lead to the next step of the cascade i.e. attack on epoxide, thus likely terminating the reaction and resulting in the formation of picrotoxic acid. We also found a literature reference with regards to the existence of picrotoxic acid as a natural product (Agrawal et al. 1999).

C

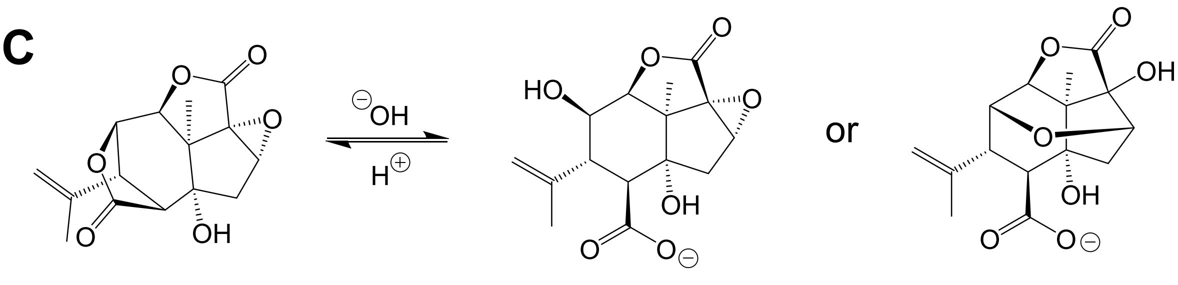

Picrotoxic acid

References

Krische MJ, Trost BM (1998) Total synthesis of methyl picrotoxate via the palladium catalyzed enyne cycloisomerization reaction. Tetrahedron 54:3693-3704.

Agarwal SK, Singh SS, Verma S, Kumar S (1999) Two picrotoxin derivatives from *Anamirta cocculus*. Phytochemistry 50:1365-1368.

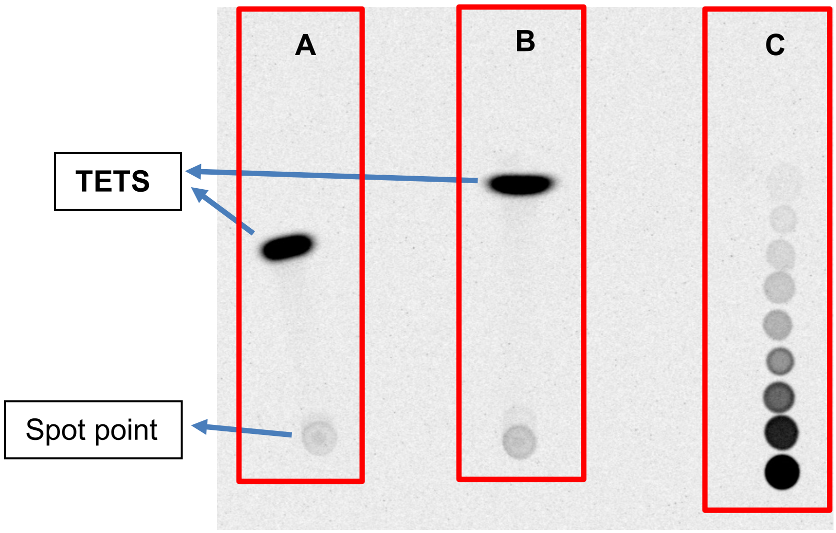

Spot intensity	Radioactivity (cpm)
23761	12
31821	26
57071	48
97976	105
133218	225
209588	429
368568	910
607123	1862
736610	3889

TETS is not metabolized during incubation with mouse liver microsomes. [^{14}C]-TETS at 152,000 cpm was incubate with mouse microsomes (male, CD1, 1 mg/mL protein) and 20 mM NADPH at 37 ºC for 1.5 hours. Two thin layer chromatography (TLC) plates were spotted with 6 µL of the resulting solution and were developed using EtOAc/hexanes = 30:70 (**A**) or EtOAc/hexanes = 50:50 (**B**) in order to visualize potential metabolites. Plates were dried, wrapped in thin plastic wrap and pressed against phosphor imaging plates for 2 days. Phosphor imaging plates were imaged using a Molecular Dynamics 9400 Typhoon phosphor imager. On both plates the intense spots with R_f values of ~0.45 (**A**) and 0.6 (**B**) correspond to 99.6% or 98.1% unmetabolized TETS. The low intensity spots at the spot point correspond to physically entrapped TETS in the protein precipitate. **C**) TLC plate with spotted standards (bottom to top: 3889, 1862, 910, 429, 225, 105, 48, 26, 12 cpm) and Table showing correlation between area intensity (corrected for background) of the spotted standards after imaging and the amount of radioactivity (corrected for background) corresponding to each spot. Radioactivity (cpm) was measured by placing equal amounts of [^{14}C]-TETS solution into liquid scintillation vials and counting on a liquid scintillation counter. Based on these reference standards, the spot point area intensities of 87356 (**A**) and 127920 (**B**) correspond to 36 and 174 cpm radioactivity or 0.4 and 1.9 % of the total radioactivity loaded on the TLC plates, respectively. The Limit of Detection (LOQ) for liquid scintillation counting was 9 × 17 = 153 cpm, where 17 cpm was the background count.

Please note that no TETS metabolites are visible in the two mobile phases.

Supplementary Figure 5
